# Lessons learned by adapting and implementing LUCHA: a deep-structure culturally tailored healthy eating randomized pilot intervention for ethnic-diverse Latinos

**DOI:** 10.3389/fpubh.2023.1269390

**Published:** 2024-02-20

**Authors:** Josiemer Mattei, Areli Caballero-González, Ana Maafs-Rodríguez, Amelia Zhang, H. June O’Neill, Cristina Gago

**Affiliations:** ^1^Department of Nutrition, Harvard TH Chan School of Public Health, Boston, MA, United States; ^2^Friedman School of Nutrition Science and Policy, Tufts University, Boston, MA, United States; ^3^Department of Population Medicine, Harvard Medical School & Harvard Pilgrim Health Care Institute, Boston, MA, United States; ^4^Department of Community Health Sciences, Boston University School of Public Health, Boston, MA, United States

**Keywords:** deep structure, cultural adaptation, cultural tailoring, nutrition education, ethnic minorities

## Abstract

**Objectives:**

To report the adaptation and implementation of LUCHA (Latinos United for a Culturally Healthy Alimentation), a pilot intervention to improve dietary quality and behaviors (primary outcomes) of Latinos in Massachusetts, US, and the lessons learned during the process, including disruptions during the COVID-19 pandemic, to help shape future programs.

**Methods:**

The cultural adaptation process was pre-planned using a framework, grounded in the Theory of Reasoned Action/Planned Behavior, and informed by formative mixed-methods research. A projected 75 self-identifying Latino adults (25–65y) were recruited with community-wide strategies and randomized to receive, in parallel, daily healthy eating text messages for 2 months, reinforced for 2 subsequent months, to either control (i.e., surface-level messages based on USDA MyPlate in Spanish), or intervention (i.e., deep-structure messages). The intervention messages were ethnically tailored to Caribbean or non-Caribbean heritages specifically, grounded in entrenched cultural attitudes, norms, and preferences. Trained research assistants administered questionnaires and clinical measurements at baseline, 2-months, and 4-months, in person (pre-pandemic) or via online video calls (at-pandemic). Clinicaltrials.gov registration #NCT04724382.

**Results:**

LUCHA faced challenges and opportunities that conveyed lessons for future cultural adaptation and implementation of healthy eating programs. Recommendations are provided to improve digital programs for diverse ethnicities, such as widening language capabilities in texting services, using familiar video call applications, and instructing participants to measure their own clinical metrics at home using guided standardized protocols.

**Conclusion:**

Tailoring nutrition programs with deep-structure cultural messages is essential when promoting healthy eating in diverse Latino heritages. LUCHA can inform programs for similar ethnic groups.

## Introduction

One of the four foundational recommendations of the United States (US) Dietary Guidelines for Americans 2020-2025 is to “*customize and enjoy nutrient-dense food and beverage choices to reflect personal preferences, cultural traditions, and budgetary considerations*” (1). The guidelines suggest including spices and herbs in place of sugars, saturated fat, and sodium, and relying on the expertise of nutrition professionals with specific cultural knowledge to healthfully prepare foods appropriate for each heritage. While this is a first step in recognizing customized dietary needs and preferences of the diverse cultures in the US, the exact path to achieve culturally appropriate healthy eating is complex and remains unpaved.

Meaningful and culturally relevant programs and interventions could support successful healthy eating behaviors and subsequent disease prevention (2). Several studies have shown higher effectiveness of dietary interventions tailored to an intended ethnic or socioeconomic group over general messages across various diet and health outcomes (3–5). However, most studies fail to report the details of the cultural adaptation, including which materials are being modified and the adaptation process. Moreover, most cultural adaptations have been limited to surface-level content, that is, observable or superficial characteristics of the intended population such as location, language, food, and appearance, rather than the deep-structure features such as embedded cultural, social, historical, environmental, and psychosocial factors that influence the behavior (6, 7). Surface-level adaptations may be well received by the group they have been adapted for, but deep-structure adaptations may be more meaningful to them, increasing their chance of receptivity, effectiveness, and maintenance. For example, a meta-analysis found that training programs for parents of ethnic minority families that had deep-structure sensitivity components were more effective in improving parenting behavior (8). It is important to detail the adaptation process of tailored behavioral interventions to inform future processes in similar populations.

Our study focused on Hispanics/Latinos (hereafter referred to as Latinos to describe individuals of all Hispanic and Latino ethnic heritages and all genders) as they are the largest ethnic group residing in the US and have a high prevalence of cardiometabolic conditions (9). Considerable variations in the prevalence of cardiometabolic conditions as well as in the food preferences and diet quality of Latinos by ethnic heritage have been well established, with individuals of Puerto Rican and Cuban (i.e., Caribbean) heritage generally having poorer diet (i.e., high intake of total energy, total fat, red and processed meat, refined grains, and sodium but low intake of vitamin C, fiber, whole grains, omega-3 fatty acids, and fruit and vegetables) and health outcomes (i.e., elevated abdominal obesity, blood pressure, and plasma glucose), compared to those of Mexican or Central and South American heritage (i.e., non-Caribbean, with generally high intake of vitamin C, calcium, fiber, fruit, poultry, fish, and whole grains, and with elevated triglycerides and low HDL-C) (9–11). However, few diet quality interventions consider the deeply rooted cultural differences that may influence attitudes toward health and diet among Latinos of diverse ethnic heritages.

This article aimed to (1) explain the deep-structure cultural adaptation of healthy eating messages for Latinos of Caribbean and non-Caribbean heritage; (2) describe the implementation of a pilot parallel intervention (LUCHA: Latinos United for a Culturally Healthy Alimentation) comparing the adapted messages to surface-level messages among Latino adults in Massachusetts, US; and (3) contribute the lessons learned during these processes with the goal of informing the design of future programs for diverse Latinos and similar populations. In March 2020, the US government declared a national emergency due to the COVID-19 pandemic (12). By then, our study was underway, and we had to modify the implementation plan; thus, we also reported the modifications made and lessons learned during the pandemic.

## Methods

### Cultural adaptation

**Figure 1:**
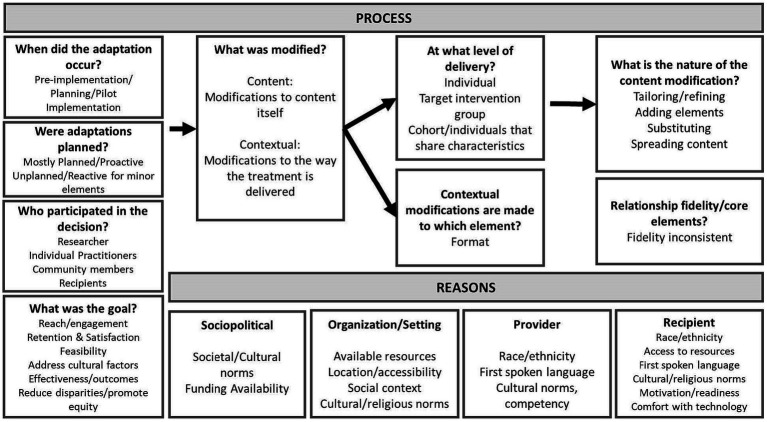
Factors for the deep-structure cultural adaptation process of healthy eating messages for Latinos, following the Framework for Reporting Adaptations and Modifications-based Implementation Strategies (with permission from Wiltsey-Stirman).

The Standard Protocol Items: Recommendations for Interventional Trials (SPIRIT) reporting guidelines on study methodology were used (13). The cultural adaptation process was proactively planned and conducted prior to LUCHA implementation using the Framework for Reporting Adaptations and Modifications-based Implementation Strategies (Figure 1) (14); minor reactive adjustments were done during implementation. Input from researchers, practitioners, community members, and participants was obtained. We identified content, context, and format of healthy eating messages at the individual, group, and heritage level, which entailed tailoring, adding, substituting, and breaking out content of the original source. The process encompassed goals across sociopolitical, organizational, provider-based, and recipient-based reasons.

The original source of healthy eating messages for US Latinos, and thus the control group in LUCHA, was the United States Department of Agriculture (USDA) MyPlate in Spanish website (15). It was selected as it is the communication initiative of the Dietary Guidelines for Americans 2020–2025 to help consumers choose healthier foods. MyPlate illustrates five food groups using a place setting, and includes online tools, containing recipes, graphics, and educational materials. To our knowledge, there is no formal cultural or linguistic adaptation of MyPlate online materials. We identified only surface-level factors in the Spanish materials: language, people, and food. For LUCHA, we copied or slightly abridged selected quotes and recipes directly from the Spanish materials, which best represented the seven selected themes for LUCHA: general nutrition knowledge, cultural aspects of healthy eating, eating habits, access and cost, cooking recipes, self-control strategies, and family meals. Direct translations of the English website were made when the quote was unavailable in Spanish. Changes to the literacy level, or tailoring at any level, were not made.

For the adapted materials (i.e., intervention group), messages were tailored guided by collected published literature and previously conducted formative research consisting of qualitative interviews with nutrition experts and Latino adults in Boston, Massachusetts, and a survey with Latino adults from the same area. Briefly, the formative exploratory sequential mixed-methods research (i.e., key informant qualitative interviews with nutrition experts, participants’ semi-structured qualitative interviews, and participants’ surveys) was grounded in the Theory of Reasoned Action/Planned Behavior (16), which also guided LUCHA design. The formative research probed for deep-structure attitudes, perceived sociocultural norms, and perceived barriers/motivators (control) that could influence behavior. The published results identified several themes regarding healthy eating (probed openly as any food or beverage participants considered beneficial to their health) that were both unique and shared among Latinos of Caribbean and non-Caribbean heritage (17). For example, most participants of all heritages agreed that healthy eating would improve health and physical appearance and that families should eat together. Deeper nuances by ethnic background were noted, though, such that the concept of ‘healthy eating’ meant limiting types of foods and nutrients and controlling portions for Caribbeans, while it meant eating wholesome and fresh foods for non-Caribbeans. For the construct of family meals, Caribbeans perceived family both as a motivator for support and caring and as a barrier (enabling unhealthy/comfort foods) to eat healthy, while non-Caribbeans focused on family members serving as role models and on the communal experience of eating. Caribbeans (vs. non-Caribbeans) were more likely to respond to statements related to the high cost of healthy foods, healthy foods as needed only for the sick, and low self-efficacy/perceived control (giving in to cravings), while non-Caribbeans were more likely to agree with indulging on special occasions. Additionally, nutrition experts identified the following deep-structure cultural factors: food as cultural identity, resignation about health, emotional regulation with food, and healthy eating as unfamiliar, expensive, and unsatisfying.

Adapted messages were created in Spanish and English by bilingual team members of diverse Latino heritages through an iterative process until consensus was attained. Healthy behaviors and concepts that were already engrained were reinforced in the messages. Misconceptions and negative perceptions of healthy eating were addressed with positive advice. When the formative research and the literature noted differences by heritage, the messages addressed these distinctions. When a topic was generally shared across Latinos, the messages were similar, while still including the deep-structure tailoring done in the first round. In addition to deep-structure components, the messages were tailored at the surface level with appropriate language, food, location, people, holidays, flavors, and strategies reported in the literature (18–20). While language is considered a surface-level factor, we tailored it more deeply as previously done for other Spanish healthy eating materials (21), by using, for example, common words for specific foods for each region or country and using the formal Spanish pronoun for “you” (*usted* rather than the informal pronoun *tú*) that is used across more countries and is considered more respectful. The linguistic readability level was set at the Flesch–Kincaid middle school grade levels. The messages included educational facts, skill-building advice, statements on cultural attitudes, and prompts for behavioral change, as framed on the “knowledge-attitude-behavior” model for health promotion (22). One adapted message was created for each of the seven selected themes and for each heritage.

Table 1 shows examples of original and adapted messages, and the deep structure and/or ethnic-specific components used for the process. For example, the Spanish MyPlate includes tips to make culturally diverse foods and cooking practices in a healthy way, such as “*using foods that are familiar to you and preparing new recipes; adding curry to chickpeas, cilantro to brown rice, or mango to your salads and smoothies*.” To deeply adapt this into a message that addressed cultural themes, we first conveyed that ‘healthy eating’ can be traditional and tasty, given the overall negative attitudes toward their taste mentioned in our formative research. Differences by ethnicity regarding the construct of ‘healthy eating’ were addressed by tailoring the message for Caribbeans around portion control and specific nutrients that concerned them, and the message for non-Caribbeans around finding wholesome, fresh foods. We concurred to include specific examples of lean meat and fish consumption in the Caribbean group, though not the non-Caribbean group, based on published prevalence of intake of these foods. We also decided to encourage non-Caribbeans to shop in Latino food markets as they had expressed familiarity and easy access to these establishments that tend to offer typical Latino food products. In an example for the cost theme, the control group received a message to “*use fresh vegetables and fruits that are in season; they are easy to obtain, have more flavor, and tend to be less expensive*.” For the adaptation, we first emphasized that food can be both healthy and affordable to address the notion among all Latinos that healthy food is expensive. We focused on fruits and vegetables to reinforce the concept among all Latinos that these are healthy foods. For ethnic tailoring, we emphasized the message on low-cost for only the Caribbean group, which markedly perceived cost as a barrier. We also highlighted low-sodium options and beans given this group’s dietary pattern. For the non-Caribbean group, we emphasized eating wholesome and fresh food from their country of origin, based on their concept of “healthy eating,” and reinforced shopping in local Latino food markets. Of note, we decided to keep the message on seasonal foods for both groups, as this concept may be unfamiliar to people relocating to the US from Latin American countries with different or less discernible agricultural seasons.

**Table 1:** Examples of healthy eating messages at the surface-level (control) and deep-structure culturally adapted (intervention) for Latinos of Caribbean and non-Caribbean ethnic heritages.

LUCHA theme	USDA MyPlate Spanish (control)	Deep-structure culturally-adapted (intervention)		Deep structure and ethnic-specific adaptation components; as informed by the literature or formative research
		Caribbean	Non-Caribbean	
General knowledge	The dietary fiber in vegetables, which are part of a healthy diet, helps reduce blood cholesterol levels and can reduce the risk of heart disease	To eat more whole grains, substitute a whole grain product for a refined product, such as eating whole grain bread instead of white bread or brown rice instead of white rice. Remember to substitute, instead of adding the whole grain product	To eat more whole grains, substitute a whole grain product for a refined product, such as eating whole wheat bread instead of white bread or whole wheat tortilla instead of flour tortilla. Remember to substitute, instead of adding the whole grain product	Rice is a staple food in Caribbean cultures Tortillas are a staple food in Mexico and Central and South American cultures Appeal to replace product rather than adding, to maintain adequate energy intake
Culture	Use foods that are familiar to you and prepare new recipes. For example, add curry to chickpeas, cilantro to brown rice, or mango to your salads and smoothies	Caribbean food can be healthy and tasty. To follow a healthier diet that includes meat, just try to make it in small quantities and buy low fat meat! Cuts such as “sirloin” or “lean” ground beef have less fat. Poultry and fish are also a good option	To find healthy foods from your country of origin that taste fresh and delicious, shop in Latino stores!	Address negative attitude among all Latinos that healthy foods are not tasty Address negative attitude among Caribbeans that traditional foods are unhealthy and have large portions Concept of “healthy eating” for Caribbeans means limiting types of foods or nutrients (such as sodium and fat) and controlling portions Concept of “healthy eating” for non-Caribbeans means eating wholesome and fresh food from their country of origin Caribbeans tend to have high intake of red and processed meats and low intake of fish
Eating habits	Eat fresh, frozen, canned or dried fruits instead of cookies, brownies or other sugary sweets	If you crave dessert, eat fresh or frozen fruit instead of ice cream or *mantecado*, cupcake, or sweet bread. You can make a strawberry shake!	When eating food that has sauce, such as enchiladas, choose a sauce that does not have cream. For example: *enchiladas verdes* instead of *enchiladas suizas*	Sweets and desserts are a top contributor to energy in Caribbeans Address perceived barrier (uncontrolled craving of unhealthy food) among Caribbeans Enchiladas are habitually consumed among non-Caribbeans
Access and cost	Use fresh vegetables and fruits that are in season. They are easy to obtain, have more flavor and tend to be less expensive	Seasonal fruits and vegetables are usually cheaper and fresher! There are healthy low-cost meals available all year: beans, cabbage, sweet potatoes or canned tomatoes low in sodium, apples and bananas	Use fresh vegetables and fruits that are in season. They are easy to obtain, are fresh like the products of your country of origin and are usually less expensive. Your local market is a great source of seasonal products	Address the notion among all Latinos that healthy food is expensive Reinforce concept among all Latinos that relate “healthy eating” with more intake of fruits and vegetables Concept of seasonality may be unfamiliar to new immigrants Cost as perceived barrier was more salient for Caribbeans Beans are staple food for Caribbeans Concept of “healthy eating” for Caribbeans means reducing sodium Concept of “healthy eating” for non-Caribbeans means eating wholesome and fresh food from their country of origin
Cooking recipes	Two-step chicken Potato salad Chocolate and yogurt cookies Light white sauce Chicken, vegetable, and brown rice Sweet and sour chicken Pasta salad French bread	Rice with pink beans Chili with beans Light fruit shake Brown rice with vegetables Oven-fried Yucca Yellow plantain with meat casserole Baked tilapia with tomatoes Grilled vegetable kabobs	Chicken Veracruz Fast and Tasty Pumpkin Flan Corn salad Turkey tacos Cod with chickpeas in Harissa sauce Green enchiladas Healthy arepa of broccoli, carrot, paprika, and cilantro Lentil soup	Concept of “healthy eating” for Caribbeans means limiting types of foods or nutrients (such as sodium and fat) and controlling portions; recipes included healthy oils, low-sodium, low sugar, whole grain versions of traditional recipes Concept of “healthy eating” for non-Caribbeans means eating fresh flavorful foods; traditional herbs and spices were emphasized in recipes
Self-control	Cook more at home to control the ingredients of your meal!	It is normal to have cravings. When you eat something unhealthy try to limit the amount. For example, if you are going to eat some potato chips, serve yourself some on a plate and close the bag so you do not eat “without thinking”	When going to a party, walk around the table to see what foods are offered before serving. Save calories with smaller portions. For example, serve yourself a palm sized amount of rice instead of a whole plate of rice	Concept of “healthy eating” for Caribbeans means limiting types of foods or nutrients and controlling portions Perceived barrier (uncontrolled craving of unhealthy food) among Caribbeans Address perceived notion of indulging at special occasions endorsed by non-Caribbeans
Family	Avoid stress at mealtime by planning a weekly menu and posting it in a location for everyone to see, like a chalkboard in the kitchen	When celebrating with the family, remember to serve yourself smaller portions to follow a healthy diet. Staying healthy is important so you can take care of them!	Families can eat healthy together! Try every week for a family member to find a healthy, delicious recipe they want to try as a family!	Concept of “family” for Caribbeans is a motivator for support and caring and a barrier (enabling unhealthy/comfort foods) to eat healthy Concept of “family” for non-Caribbeans is being a role model and having a communal experience when eating (around food)

### Study design

**Figure 2:**
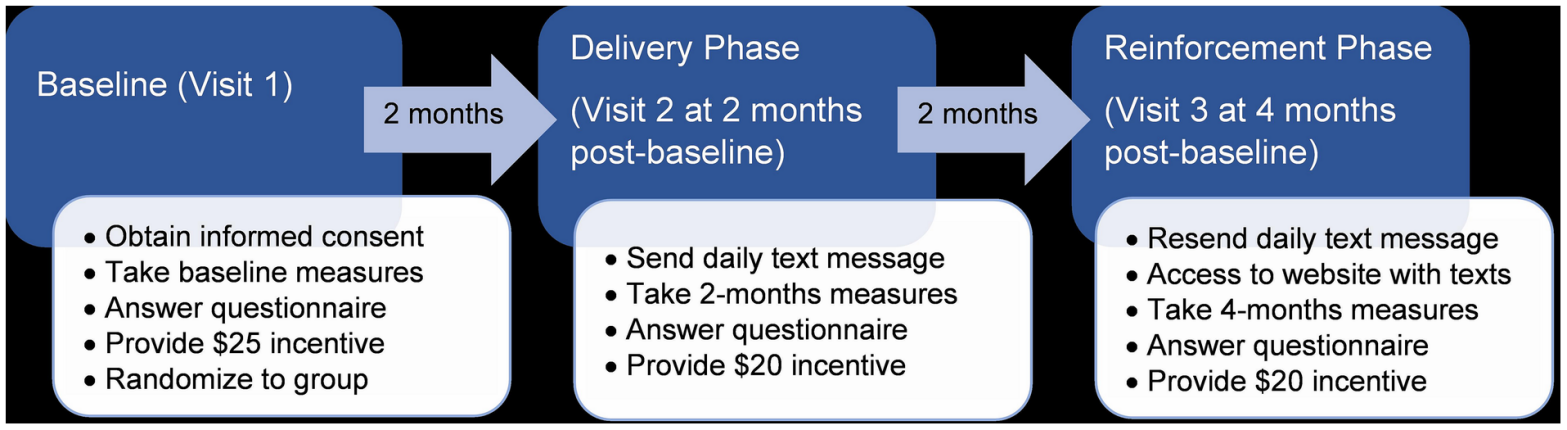
Protocol flowchart of Latinos United for a Culturally Healthy Alimentation: a pilot intervention of deep-structure culturally-adapted vs. surface-level healthy eating messages for Latinos in Massachusetts, US.

LUCHA was designed as a parallel two-arm double-blind randomized pilot and feasibility intervention. The protocol included three visits to administer interviews and collect clinical measurements for intervention and control participants: one at baseline, 2 months, and 4 months. After the baseline visit, eligible participants received daily healthy eating messages sent via text messages for 2 months (delivery phase); after the two-month visit, participants received the same texts again for another two more months and were also given access to the messages on the study website (reinforcement phase) (Figure 2). The control group received surface-level healthy eating messages, while the intervention group received deep-structure messages tailored to their predominant ethnic heritage of Caribbean (e.g., Puerto Rico, Dominican Republic, and Cuba) or non-Caribbean (e.g., Mexico, Central and South America). Each visit was estimated to take about 1-2 h to complete. All participants remained enrolled in the study for its duration unless they were removed from the study, they actively dropped out, or we lost contact. The Institutional Review Board (IRB) of Harvard TH Chan School of Public Health approved this study. LUCHA was registered at clinicaltrials.gov under the identification number NCT04724382.

LUCHA was launched in April 2019 in partnership with community clinics in Boston, Massachusetts, US neighborhoods with high concentrations of Latino residents and Latino-serving organizations. LUCHA had to pause in-person activities on March 2020 due to the COVID-19 pandemic. During a 4-months pause, study protocols were limited to administering pending questionnaires to enrolled participants and asking optional questions on signs, symptoms, management, and behaviors during COVID-19 via online or video platforms; culturally relevant and trustworthy information on COVID-19 from trustworthy national organizations was also provided. Text-based messages were delivered daily as scheduled during this time. On July 2020, LUCHA resumed with changes to the protocol, until completion on September 2021. The Methods describe the protocols as originally designed (denoted as “pre-pandemic”), as well as the changes made in response to the COVID-19 restrictions (denoted as “at-pandemic”). Other changes made to the study during its course that were not in direct response to COVID-19 are described in the Results section as part of the lessons learned.

### Recruitment

#### Pre-pandemic

Research assistants with proper identification approached individuals in public areas of the partner clinics to inform them about the study. Informational flyers were posted in the partner clinics, in Latino-serving community organizations, and in public sites of Latino neighborhoods in the Greater Boston area such as public housing, churches, service shops, markets, food establishments, fairs, festivals, and parking lots. For referrals, participants were asked to give a study flyer to individuals currently not living in the same household. Eligibility criteria included self-identifying Hispanic/Latino individuals aged 25–65y, who had resided in the Greater Boston area for at least the previous 6 months and were not planning to move for the next 6 months, and who had access to a cellphone capable of receiving calls and text messages for the duration of the study. Exclusions included a self-reported diagnosis of cancer, diabetes, gastrointestinal disease, coronary heart disease, stroke or heart attack, severe dietary allergies or restrictions, current pregnancy, or institutionalization. Eligible participants provided signed informed consent.

#### At-pandemic

Recruitment switched primarily to Facebook (posting on public Latino-oriented community and business pages and groups), with some passive community approaches (i.e., posting flyers) using public health safety precautions. Eligibility criteria remained the same except that the area of residency was expanded to the state of Massachusetts, as social media and video calls (for appointments) allowed for wider recruitment and implementation. Participants recruited at-pandemic provided oral informed consent.

#### Both pre- and at-pandemic

Interested individuals contacted the study by phone call, email, or text to be screened for potential eligibility by a bilingual (English/Spanish) research assistant. Eligible individuals were scheduled for a baseline interview, where informed consent was obtained from all individual participants included in the study. Flexible times and days for appointments were available; completion by phone was an option. At the completion of the baseline interview, participants were randomized to a group via simple probability sampling. Random numbers were generated using Microsoft Excel within the range of 0–1, and at the time of assignment, if the subsequent number fell at or below 0.5, the participant was assigned to the control group. If the number was greater than or equal to 0.5, the participant was assigned to the intervention group according to their heritage (i.e., intervention for Caribbeans or intervention for non-Caribbeans). Participants were compensated for their time up to $65, such that they received $25 at baseline, and $20 at each remaining visit through physical gift cards pre-pandemic, and electronic gift cards or physical gift cards sent via certified mail at-pandemic. Reimbursement for transportation was available upon request during pre-pandemic.

To assist with attendance and retention, participants were contacted up to five times to schedule or remind them of the appointments using their preferred method of communication. Three points of contact were recorded to reach participants if not responsive. To assist with data completion, participants were allowed to pause the interviews at any time and resume them within the subsequent 14 days; they were contacted up to five times to remind them to complete the pending questions. Process evaluation (i.e., reach of texts; fidelity of texting delivery and receipt; rates of recruitment, rescheduling, retention, adverse events, data quality checks, etc.) was monitored bi-weekly and adjustments were implemented as needed.

### Data collection

Research assistants were trained and re-trained on questionnaire implementation, uniform data collection and processing, cultural sensitivity, treatment fidelity, and confidentiality. All personnel in contact with participants was blinded to group allocation, except for one research assistant who exclusively sent text messages and mailed letters with information on the group-dependent website. To assist with double blinding, participants were informed that they would receive messages on healthy eating, without further details on the type of messages.

#### Pre-pandemic

Data collection and procedures were conducted in a single private room in the partner clinics or at Harvard TH Chan School of Public Health by bilingual research assistants. When scheduling an interview, participants were instructed to wear light clothing for the body measurements. At each visit, the research assistant measured the participant’s waist and hip circumference to the nearest 0.1 cm using a stretch-resistant measuring tape, and weight to the nearest 0.1 kg using a Detecto SlimTalkXL scale (Detecto, Webb City, MO) following standard procedures. After sitting for a 5-min rest, the participant was measured for blood pressure at either arm using an Omron 10 Series Upper Arm Blood Pressure Monitor (Omron, Kyoto, Japan). Instruments were tested and calibrated before and during the study. Measurements were repeated thrice, and an average value was calculated across all three measurements. Height was self-reported. A letter with these values was sent to the participant upon request.

At each visit, a research assistant administered a questionnaire in the participant’s preferred language. Answers were entered using the real-time web-based electronic data capture tool, “Research Electronic Data Capture” (REDCap) (23). Participants received a light, healthy snack, and water.

The questionnaire included sections to assess our primary and secondary outcomes (described below), as well as single-item question on age, sex-at-birth, education, income, work history, health care, medical diagnoses, food security and assistance, smoking, alcohol use, physical activity, and sleep quantity and quality. The questionnaire also included psychosocial measures previously validated among Spanish speakers: a 10-item perceived stress scale (24), a 3-item loneliness scale (25), the 20-item Center for Epidemiologic Studies Depression Scale (26), and the Interpersonal Support Evaluation List-12 for social support (27).

The primary outcomes were changes in dietary intake (i.e., quality) and behaviors, at 2-months and 4-months. Diet quality was measured using an adapted brief diet quality screener that probed frequency of intake of 18 major food groups and has been validated against 24-h recalls (*r* = 0.61) and various nutrients (28). Briefly, standard portions are described to the participant, who is then asked to report the consumption in the past month of one daily portion of bread, vegetables, fruit, milk/yogurt, rice/pasta, oils (corn, sunflower or olive), or breakfast cereal; 4–6 portions per week of meat, sausage, cheese, sweets/desserts, butter/fat, other oils, or fast food; and 2–3 portions per week of fish, legumes (e.g., beans), or nuts. Intake in the indicated frequency for each of these foods is allocated 2 points; intake at a higher frequency is given 3 points, and at a lower frequency is given 1 point; except for meat, sausage, cheese, sweets/desserts, butter/fat, other oils, or fast food that are reverse-coded. Daily consumption of one alcoholic drink is scored as 3; lower and higher intakes are scored 1. The food items are added for a total possible score range of 18–54.

The dietary behaviors measured were food consumption away from home, mealtime habits with the family, cooking practices (such as portion or nutrient control), and nutrition awareness (such as knowledge of the USDA My Plate or nutrition facts label). Food consumption away from home was probed using a questionnaire administered in the Hispanic Community Health Study/Study of Latinos (29). The remaining constructs were measured via questions adapted from the Food Attitudes and Behaviors Survey of the National Cancer Institute (Cronbach’s α coefficient ≥ 0.68) (30, 31) and a validated dietary behaviors questionnaire for Latinos (Cronbach’s α coefficients 0.47–0.48) (32). The questions have been subsequently applied in studies with participants of Latino heritage (33, 34).

Secondary outcomes included changes in the 45-item Diet Satisfaction Questionnaire to assess healthy lifestyle, cost, convenience, family dynamics, preoccupation with food, negative aspects, and planning and preparation (35); cultural dietary attitudes and perceptions from the survey developed during formative research (17); emotional, uncontrolled and restrictive eating behaviors using the Three-Factor Eating Questionnaire (36); and an adapted Fulkerson home food inventory (37). Additionally, we evaluated program satisfaction and engagement by asking participants whether they agreed or disagreed with various statements regarding the program components and implementation.

#### At-pandemic

Interviews shifted to the IRB-approved password-protected online video platforms (i.e., Zoom or Microsoft Teams). Phone calls were also available via a password protected Google Voice account. Participants were asked the same questions as pre-pandemic. They were mailed detailed instructions, with diagrams, on how to take their waist and hip circumference and blood pressure at home as used in the pre-pandemic protocols. We mailed a measuring tape and a wrist blood pressure monitor [G.LAB md1520/2222/2231 (Fremont, CA) or LifeSource UB-521 (Mississauga, ON)] directly to the participant’s home. Participants kept these instruments after the program ended. The research assistant guided the participant on how to use the instruments and visually confirmed the measurements by showing the instrument or a picture during the video call or texting a picture or the values if not using video platforms. Sending weight scales was not viable at the time of protocol changes, thus, participants were asked to self-report their weight.

### Text messages and website access

Text messaging was chosen given its high acceptability and feasibility among Latinos, and efficacy of previous interventions for healthy eating in this population (38–40). Texting also broadens access for populations without a smartphone or internet. Daily text messages (56 in total, over 2 months) were delivered to participants in each assigned group in their preferred language throughout the initial 2 months (delivery phase). After the delivery phase, an unblinded research assistant provided a letter to the participant with detailed instructions on how to log into a password-protected Harvard University-hosted website that contained all the text messages specific to the participant’s assigned group. Because prior research has demonstrated periodic reminders may be effective in diet behavior interventions (41), we designed a reinforcement phase where participants received the same 56 text messages during the subsequent 2 months.

The fee-based automated Short Message Servicing (SMS) platform EZTexting was initially selected to deliver the text messages, but because of its limited Spanish language capabilities at the time of the study (e.g., accents), the team promptly switched to Google Voice (an SMS platform free at the time of the study) to send messages in both Spanish and English. Participants had the opportunity to send messages with questions or comments to the study using Google Voice. These messages were answered by a research assistant; consultation with the team was sought if needed.

### Sample size and projected analysis

The projected sample size was 75 participants based on estimates for pilot studies, with a 0.05 Type I error, 80% power, and assumed 20% dropout rate based on previous projects in this population (42, 43). Primary analysis will be based on intention-to-treat. Per protocol analysis will also be done. We will use repeated measure analyses to test differences in mean change in 2-mo and 4-mo (from baseline) in primary outcomes for control vs. intervention groups, adjusting for baseline score, and any characteristic that was not randomly allocated. Secondary analyses will test similar changes by Caribbean vs. non-Caribbean heritage, and for secondary outcomes. Data analysis will follow the Consolidated Standards of Reporting Trials (CONSORT) guidelines (44).

## Results

The March 2020 emergency of COVID-19 triggered protocol changes that imparted lessons on study implementation. At-pandemic, the video platforms approved by IRB for interviews were unfamiliar or unavailable to several participants and sometimes required registration or a fee; participants, instead, often requested video calls through popular free social media platforms or apps that they already used for casual communication, such as FaceTime, WhatsApp, or Facebook Messenger. Limited access to internet connectivity (reliable or at all), or to a computer with video capability, was also commonly mentioned by participants, who preferred phone-based options. Despite this preference and need, IRB declined approval of the more familiar online video platforms.

Because the measurement instruments were mailed directly to the participant’s home, the study personnel could not test or calibrate them before use. The additional steps in guiding the participant on how to set up and use the instruments during the video call, lengthened the duration of the interview. Mailing letters, gift cards, and materials increased costs—by approximately US$10 (letters and gift cards) to US$30 (materials) per participant—and delayed the protocol timeline by 5–10 days per participant. Several deliveries were lost, and 7 wrist monitors were reported defective and re-sent, which further delayed the interviews and increased costs.

General lessons were also learned throughout the whole study period, especially relevant for studies delivered digitally. After the text delivery phase, nearly all participants needed help with the process of accessing the password-protected website that contained all the text messages specific to their assigned group. As the institutional website was cumbersome to access and the password was difficult to remember and could not be changed; this guidance required extra time and effort from the interviewers and participants. Another technological challenge was that Google Voice was not automatized. Therefore, an unblinded research assistant needed to manually send messages to each participant daily, which was time consuming and prone to lapses to fidelity of protocol. Pictures or long texts (e.g., recipes) sent through Multimedia Messaging Service (MMS) were received as small and unclear on some cellphones, and sometimes were received in the wrong order, even when sent correctly, confusing the participants. Thus, MMS had to be limited and recipes were instead sent in multiple text messages or as a link, which was cumbersome and may have excluded access for those without internet connection.

## Discussion

LUCHA demonstrates that creating deep-structure culturally tailored nutrition messages to specific Latino ethnic heritages is feasible and valuable. Although gaps remain in methodological approaches to create such messages, recent interest from the Dietary Guideline for Americans in encouraging culturally appropriate healthy diets should pave the way for more and better designed deep-structure dietary programs and interventions, especially for underserved ethnic groups. Such endeavors are starting to emerge and should be emulated, including the deep-structure modifications made to the culturally adapted version of the “Coping with Stress Course” (into the Resilient in spite of Stressful Events) for Black adolescents in a low-income urban community, and the self-help program “Step-by-Step” for psychological distress for Albanian-speaking immigrants in Switzerland and Germany (45, 46).

**Figure 3:**
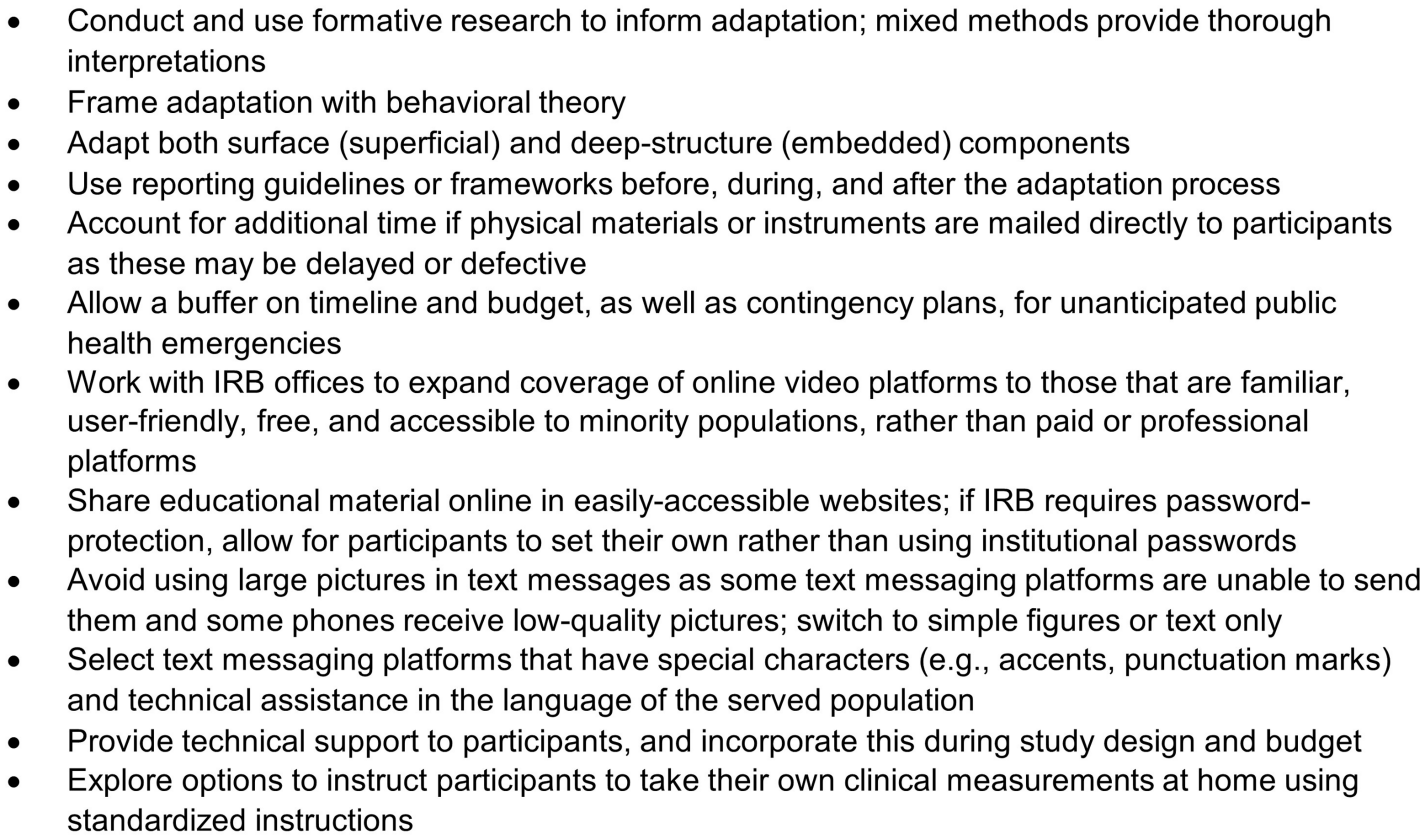
Recommendations for cultural adaptation of text-message-based behavioral programs for ethnic groups.

We learned several key lessons while adapting and implementing LUCHA, which has encouraged us to propose recommendations for future research and practice (Figure 3). First, formative research, especially those employing mixed-methods design (47), is essential in informing cultural adaptations. Quantitative data may help collect metrics of outcomes generalizable to the intended population while qualitative data may help contextualize such data in a meaningful manner. During the adaptation process, researchers should apply an evidence-based behavioral theory that aligns with their research question, include both surface and deep-structure components, and record their process using reporting guidelines. Additional time, budget, and contingency plans should also be prepared in case of delayed or defective instruments mailed to participants, or the unexpected onset of public health emergencies (i.e., COVID-19). We also faced institutional barriers with IRB that required password-protected professional platforms for the online video interviews and the website where materials were shared, limiting access to participants who were unfamiliar or unable to open such. Latinos continue to be underrepresented in research and several strategies have been identified to prioritize and facilitate their recruitment and retention in biomedical studies. To overcome obstacles that would hinder their representation, IRB offices should consider alternatives for familiar, free, and user-friendly online platforms, especially in a post-COVID-19 era that has shifted many research protocols online. Providing technical support to participants should be part of the study planning and budgeting. Researchers and practitioners delivering text-based health interventions should opt for content quality over intricate visuals for ease of delivery and select text messaging platforms with services in the language of the served population. Updated and new platforms should solve these barriers. Lastly, researchers may consider instructing participants to measure their own clinical values at home using standardized protocols and guided via video; doing so may help empower participants and bolster health behaviors, as has been previously shown with improvements in blood pressure control, self-care, and self-efficacy (48).

This study has some limitations. Creating materials for LUCHA was specific to the Latino population in Massachusetts and may not be generalizable to other sites in the US or globally. Messages were designed for Caribbean vs. non-Caribbean groups that may be homogenous in some ways, but the content may not reflect cultural nuances of distinct ethnic groups by country of origin. Future formative research and interventions should consider these intricacies. However, the same cultural tailoring methodology utilized in this study may be applied in other settings and for different ethnic groups. Furthermore, this study was completed during the height of the COVID-19 pandemic, which created a unique environment that may not be replicated in other studies. However, the pandemic granted an opportunity to redesign the project and, subsequently, learn useful lessons for future research.

LUCHA supports the tailoring of nutrition programs with deep-structure cultural messages as an essential step when promoting healthy diets in Latinos of diverse ethnic heritages. Recommendations from our adaptation and implementation process can inform similar programs for other ethnic groups.

## Data availability statement

The original contributions presented in the study are included in the article/supplementary material, further inquiries can be directed to the corresponding author.

## Ethics statement

The studies involving humans were approved by Institutional Review Board of Harvard TH Chan School of Public Health. The studies were conducted in accordance with the local legislation and institutional requirements. The participants provided their written informed consent to participate in this study.

## Author contributions

JM: Conceptualization, Data curation, Funding acquisition, Investigation, Methodology, Project administration, Supervision, Visualization, Writing – original draft, Writing – review & editing, Formal analysis. AC-G: Data curation, Formal analysis, Investigation, Visualization, Writing – original draft, Writing – review & editing. AM-R: Data curation, Formal analysis, Investigation, Visualization, Writing – original draft, Writing – review & editing, Methodology. AZ: Data curation, Writing – review & editing, Methodology. HJO’N: Data curation, Formal analysis, Visualization, Writing – review & editing, Methodology. CG: Data curation, Investigation, Writing – review & editing, Methodology.

## References

[1] Dietary Guidelines for Americans (2020). Available at: https://www.dietaryguidelines.gov/ (Accessed June 28, 2022).

[2] Winham DM. Culturally tailored foods and CVD prevention. Am J Lifestyle Med. (2009) 3:64S–8S. doi: 10.1177/1559827609335552, PMID: 20046905 PMC2782861

[3] Nierkens V Hartman MA Nicolaou M Vissenberg C Beune EJAJ Hosper K. Effectiveness of cultural adaptations of interventions aimed at smoking cessation, diet, and/or physical activity in ethnic minorities. a systematic review. PLoS One. (2013) 8:e73373. doi: 10.1371/journal.pone.0073373, PMID: 24116000 PMC3792111

[4] Hammons AJ Hannon BA Teran-Garcia M Barragan M Villegas E Wiley A. Effects of culturally tailored nutrition education on dietary quality of hispanic mothers: a randomized control trial. J Nutr Educ Behav. (2019) 51:1168–76. doi: 10.1016/j.jneb.2019.06.017, PMID: 31375361

[5] Risica PM Strolla LO Fournier L Kirtania U Upegui D Zhao J. Effectiveness of different methods for delivering tailored nutrition education to low income, ethnically diverse adults. Int J Behav Nutr Phys Act. (2009) 6:24. doi: 10.1186/1479-5868-6-24, PMID: 19416525 PMC2688475

[6] Broyles SL Brennan JJ Herzog Burke K Kozo J Taras HL. Cultural adaptation of a nutrition education curriculum for latino families to promote acceptance. J Nutr Educ Behav. (2011) 43:S158–61. doi: 10.1016/j.jneb.2011.02.014, PMID: 21683288 PMC3124678

[7] Resnicow K Baranowski T Ahluwalia JS Braithwaite RL. Cultural sensitivity in public health: Defined and demystified. Ethn Dis. (1999) 9:10–21. PMID: 10355471

[8] van Mourik K Crone MR de Wolff MS Reis R. Parent training programs for ethnic minorities: a meta-analysis of adaptations and effect. Prev Sci. (2017) 18:95–105. doi: 10.1007/s11121-016-0733-5, PMID: 27882498 PMC5236066

[9] Heiss G Snyder ML Teng Y Schneiderman N Llabre MM Cowie C. Prevalence of metabolic syndrome among hispanics/latinos of diverse background: The Hispanic Community Health Study/Study of Latinos. Diabetes Care. (2014) 37:2391–9. doi: 10.2337/dc13-2505, PMID: 25061141 PMC4113166

[10] Siega-Riz AM Sotres-Alvarez D Ayala GX Ginsberg M Himes JH Liu K. Food-group and nutrient-density intakes by Hispanic and Latino backgrounds in the Hispanic Community Health Study/Study of Latinos. Am J Clin Nutr. (2014) 99:1487–98. doi: 10.3945/AJCN.113.082685, PMID: 24760972 PMC4021787

[11] Mattei J Sotres-Alvarez D Daviglus ML Gallo LC Gellman M Hu FB. Diet quality and its association with cardiometabolic risk factors vary by hispanic and latino ethnic background in the hispanic community health study/study of latinos. J Nutr. (2016) 146:2035–44. doi: 10.3945/JN.116.231209 27605403 PMC5037869

[12] Federal Register: Declaring a National Emergency Concerning the Novel Coronavirus Disease (COVID-19) Outbreak (2020). Available at: https://www.federalregister.gov/documents/2020/03/18/2020-05794/declaring-a-national-emergency-concerning-the-novel-coronavirus-disease-covid-19-outbreak (Accessed November 12, 2023).

[13] Chan AW Tetzlaff JM Gøtzsche PC Altman DG Mann H Berlin JA. SPIRIT explanation and elaboration: guidance for protocols of clinical trials. BMJ. (2013) 346:e7586. doi: 10.1136/BMJ.E7586 23303884 PMC3541470

[14] Miller CJ Barnett ML Baumann AA Gutner CA Wiltsey-Stirman S. The FRAME-IS: a framework for documenting modifications to implementation strategies in healthcare. Implement Sci. (2021) 16:36. doi: 10.1186/S13012-021-01105-3, PMID: 33827716 PMC8024675

[15] En español | MyPlate. Available at: https://www.myplate.gov/resources/en-espanol (Accessed June 28, 2022).

[16] Sutton S. Health behavior psychosocial theories In: International Encyclopedia of the Social and Behavioral Sciences. 2nd ed (2015)

[17] Caballero-Gonzalez A Lopez-Cepero A Mattei J. Deep-structure attitudes and reasons towards healthy eating and self-rated diet and health in ethnically diverse US-Hispanics/Latinos. Health Promot Pract. (2023). doi: 10.1177/15248399231214968 PMC13502142 38102803

[18] McCurley JL Gutierrez AP Gallo LC. Diabetes prevention in U.S. hispanic adults: a systematic review of culturally tailored interventions. Am J Prev Med. (2017) 52:519–29. doi: 10.1016/j.amepre.2016.10.028 27989451 PMC5362335

[19] Stotz S Habibi M Sanville L Cotto-Rivera E Soler A Powell A. Adapting a nutrition education curriculum for spanish-speaking adults experiencing low-income: recommendations from key stakeholders. Ecol Food Nutr. (2021) 60:737–50. doi: 10.1080/03670244.2021.1899917, PMID: 33781137

[20] Mier N Ory MG Medina AA. Anatomy of culturally sensitive interventions promoting nutrition and exercise in hispanics: a critical examination of existing literature. Health Promot Pract. (2007) 11:541–54. doi: 10.1177/1524839908328991, PMID: 19193933 PMC3780354

[21] Maafs-Rodríguez A Otis B Mattei J. Cultural adaptation and social media promotion of healthy eating guides for spanish speakers. J Nutr Educ Behav. (2022) 54:863–71. doi: 10.1016/j.jneb.2022.03.008, PMID: 35750617

[22] Bettinghaus EP. Health promotion and the knowledge-attitude-behavior continuum. Prev Med (Baltim). (1986) 15:475–91. doi: 10.1016/0091-7435(86)90025-3, PMID: 3774779

[23] Harris PA Taylor R Thielke R Payne J Gonzalez N Conde JG. Research electronic data capture (REDCap)-A metadata-driven methodology and workflow process for providing translational research informatics support. J Biomed Inform. (2009) 42:377–81. doi: 10.1016/j.jbi.2008.08.010 18929686 PMC2700030

[24] Perera MJ Brintz CE Birnbaum-Weitzman O Penedo FJ Gallo LC Gonzalez P. Factor structure of the Perceived Stress Scale-10 (PSS) across English and Spanish language responders in the HCHS/SOL Sociocultural Ancillary Study. Psychol Assess. (2017) 29:320–8. doi: 10.1037/PAS0000336, PMID: 27280744 PMC5148735

[25] Hughes ME Waite LJ Hawkley LC Cacioppo JT. A short scale for measuring loneliness in large surveys: results from two population-based studies. Res Aging. (2004) 26:655–72. doi: 10.1177/0164027504268574, PMID: 18504506 PMC2394670

[26] Soler J Pérez-Sola V Puigdemont D Pérez-Blanco J Figueres MAE. Estudio de validación del Center for Epidemiologic Studies-Depresion (CES-D) en una población española de pacientes con trastornos afectivos Validation study of the Center for Epidemiological Studies-Depression of a Spanish population of patients with af. Actas Luso Esp Neurol Psiquiatr Cienc Afines. (1997) 25:243–9. PMID: 9412163

[27] Merz EL Roesch SC Malcarne VL Penedo FJ Llabre MM Weitzman OB. Validation of interpersonal support evaluation list-12 (ISEL-12) scores among English-and Spanish-Speaking Hispanics/Latinos from the HCHS/SOL sociocultural ancillary study. Psychol Assess. (2014) 26:384–94. doi: 10.1037/A0035248, PMID: 24320763 PMC4048059

[28] Schröder H Benitez Arciniega A Soler C Covas MI Baena-Díez JM Marrugat J. Validity of two short screeners for diet quality in time-limited Settings. Public Health Nutr. (2012) 15:618–26. doi: 10.1017/S1368980011001923, PMID: 21859517

[29] McClain AC Ayala GX Sotres-Alvarez D Siega-Riz AM Kaplan RC Gellman MD. Frequency of intake and type of away-from-home foods consumed are associated with diet quality in the hispanic community health study/study of latinos (HCHS/SOL). J Nutr. (2018) 148:453–63. doi: 10.1093/JN/NXX067, PMID: 29546313 PMC6251533

[30] Food Attitudes and Behaviors (FAB). Division of Cancer Control and Population Sciences (DCCPS). Available at: https://cancercontrol.cancer.gov/brp/hbrb/food-attitudes-and-behaviors (Accessed November 12, 2023).

[31] Erinosho TO Pinard CA Nebeling LC Moser RP Shaikh AR Resnicow K. Development and implementation of the National Cancer Institute’s food attitudes and behaviors survey to assess correlates of fruit and vegetable intake in adults. PLoS One. (2015) 10:e0115017. doi: 10.1371/JOURNAL.PONE.0115017, PMID: 25706120 PMC4338082

[32] Fernandez S Olendzki B Rosal MC. A dietary behaviors measure for use with low-income, Spanish-speaking Caribbean Latinos with type 2 diabetes: The Latino Dietary Behaviors Questionnaire (LDBQ). J Am Diet Assoc. (2011) 111:589–99. doi: 10.1016/J.JADA.2011.01.015, PMID: 21443994 PMC3148606

[33] Bezares N McClain AC Tamez M Rodriguez-Orengo JF Tucker KL Mattei J. Consumption of foods away from home is associated with lower diet quality among adults living in puerto rico. J Acad Nutr Diet. (2023) 123:95–108.e10. doi: 10.1016/J.JAND.2022.06.009 35738537 PMC9763551

[34] López-Cepero A Tucker KL Rodríguez-Orengo JF Mattei J. Self-reported engagement in healthy eating behaviors is associated with favorable dietary intake among adults in Puerto Rico. Nutr Res. (2023) 118:137–45. doi: 10.1016/J.NUTRES.2023.07.011, PMID: 37666009 PMC10592052

[35] James BL Loken E Roe LS Myrissa K Lawton CL Dye L. Validation of the Diet Satisfaction Questionnaire: a new measure of satisfaction with diets for weight management. Obes Sci Pract. (2018) 4:506–14. doi: 10.1002/osp4.299, PMID: 30574344 PMC6298208

[36] Cappelleri JC Bushmakin AG Gerber RA Leidy NK Sexton CC Lowe MR. Psychometric analysis of the Three-Factor Eating Questionnaire-R21: Results from a large diverse sample of obese and non-obese participants. Int J Obes. (2009) 33:611–20. doi: 10.1038/ijo.2009.74, PMID: 19399021

[37] Fulkerson JA Nelson MC Lytle L Moe S Heitzler C Pasch KE. The validation of a home food inventory. Int J Behav Nutr Phys Act. (2008) 5:55. doi: 10.1186/1479-5868-5-55 18983668 PMC2587472

[38] Santo K Hyun K de Keizer L Thiagalingam A Hillis GS Chalmers J. The effects of a lifestyle-focused text-messaging intervention on adherence to dietary guideline recommendations in patients with coronary heart disease: An analysis of the TEXT ME study. Int J Behav Nutr Phys Act. (2018) 15:45. doi: 10.1186/s12966-018-0677-1 29792202 PMC5967045

[39] Jin Q Boyce TW Kang H Nervi L Sussman AL Guest DD. Acceptability of phone calls and texts to promote healthy behaviors among spanish-speaking hispanics. Hisp J Behav Sci. (2021) 43:278–93. doi: 10.1177/07399863211034950

[40] Hall AK Cole-Lewis H Bernhardt JM. Mobile text messaging for health: A systematic review of reviews. Annu Rev Public Health. (2015) 36:393–415. doi: 10.1146/annurev-publhealth-031914-122855, PMID: 25785892 PMC4406229

[41] Fry JP Neff RA. Periodic prompts and reminders in health promotion and health behavior interventions: Systematic review. J Med Internet Res. (2009) 11:e16. doi: 10.2196/jmir.1138, PMID: 19632970 PMC2762806

[42] Johanson GA Brooks GP. Initial scale development: sample size for pilot. Studies. (2009) 70:394–400. doi: 10.1177/0013164409355692

[43] Hertzog MA. Considerations in determining sample size for pilot studies. Res Nurs Health. (2008) 31:180–91. doi: 10.1002/NUR.20247 18183564

[44] Schulz KF Altman DG Moher D. CONSORT 2010 statement: Updated guidelines for reporting parallel group randomised trials. BMJ. (2010) 1:100–7. doi: 10.4103/0976-500X.72352 PMC3043330 21350618

[45] Heim E Burchert S Shala M Kaufmann M Cerga Pashoja A Morina N. Effect of cultural adaptation of a smartphone-based self-help programme on its acceptability and efficacy: study protocol for a randomized controlled trial | PsychArchives. (2020). Available at: https://www.psycharchives.org/en/item/838b68b4-363f-4409-aa8e-c8d3e059e32a (Accessed June 28, 2022). 10.32872/cpe.2743 PMC11303917 39119053

[46] Clarke AT Soto GE Cook J Iloanusi C Akwarandu A Still PV. Adaptation of the coping with stress course for black adolescents in low-income communities: examples of surface structure and deep structure cultural adaptations. Cogn Behav Pract. (2021) 29:738–49. doi: 10.1016/j.cbpra.2021.04.005, PMID: 36387782 PMC9642973

[47] Strolla LO Gans KM Risica PM. Using qualitative and quantitative formative research to develop tailored nutrition intervention materials for a diverse low-income audience. Health Educ Res. (2006) 21:465–76. doi: 10.1093/her/cyh072, PMID: 16303783

[48] Avegno KS Roberson KB Onsomu EO Edwards MF Dean EL Bertoni AG. Evaluating a telephone and home blood pressure monitoring intervention to improve blood pressure control and self-care behaviors in adults with low-socioeconomic status. Int J Environ Res Public Health. (2023) 20:5287. doi: 10.3390/IJERPH20075287 37047903 PMC10094475

